# Pypes: Workflows for Processing Multimodal Neuroimaging Data

**DOI:** 10.3389/fninf.2017.00025

**Published:** 2017-04-11

**Authors:** Alexandre M. Savio, Michael Schutte, Manuel Graña, Igor Yakushev

**Affiliations:** ^1^Department of Nuclear Medicine, Klinikum Rechts der Isar, Technische Universität MünchenMunich, Germany; ^2^Asociación Python San Sebastián (ACPySS)San Sebastián, Spain; ^3^Computational Intelligence Group, University of the Basque Country (UPV/EHU)San Sebastián, Spain; ^4^Neuroimaging Center at Technische Universität München (TUM-NIC), Technische Universität MünchenMunich, Germany

**Keywords:** python, brain imaging, PET, MRI, registration, denoising, image analysis, brain connectivity

## 1. Introduction

Every year, enormous amounts of scientific data are made available to the public (Poline et al., 2012). This trend is due to an increasing demand for transparency, efficiency, and reproducibility. Neuroimaging is a salient example of this trend.

In response to the growing concern about the need of publishing relevant software codes (Ince et al., 2012) in the context of results' reproducibility, there is an increasing number of open source initiatives that support code distribution and co-development (Halchenko and Hanke, 2012). The growing diversity of imaging modalities demand from the practitioner a deep technical knowledge of data pre- and post-processing. Consequently, there are open and free tools facilitating image data analysis, e.g., the Python module Nipype^1^. It offers a homogeneous programming interface and integrates many of these data processing tools. In this sense, resting-state functional magnetic resonance imaging (rsfMRI) is receiving considerable attention by the community with tools such as the Configurable Pipeline for the Analysis of Connectomes (C-PAC)^2^, and the Data Processing Assistant for Resting-State fMRI (DPARSF)^3^.

As a further contribution to this development, this paper presents a new Python module Pypes—https://github.com/Neurita/pypes. It includes a collection of workflows, reusable neuroimaging pipelines using Nipype, along with some utilities. This library seeks to simplify the reusability and reproducibility of multimodal neuroimaging studies, offering pre- and post-processing utilities inspired by C-PAC. It pre-processes Positron Emission Tomography (PET) and three MRI-based modalities: structural, rsfMRI, and diffusion-tensor MRI (DTI). It also shares an easy-to-use pipeline for COBRE^4^, a public available dataset. Pypes has been motivated by a need for efficient and reproduceable brain PET/MRI data processing methods. Namely, hybrid PET/MRI scanners become a relevant source of multimodal imaging data, posing new computational challenges. For instance, a simultaneous measurement of brain glucose metabolism and functional connectivity (Aiello et al., 2015; Riedl et al., 2016) opens new perspectives in neuroscience. Structural, functional, and metabolic imaging protocols have been proposed for clinical evaluation of dementia and neuro-oncological cases (Werner et al., 2015; Henriksen et al., 2016). Pypes' immediate motivation was to process PET/MRI data from an ongoing study with more than 400 subjects with suspected neurodegenerative disorders.

The paper is organized as follows. After introducing the Python neuroimaging ecosystem and specifically Nipype, we show how to prepare image data for the workflows available in Pypes. Then, we describe worflow configuration for specific imaging modalities. Finally, we present the Pypes pre-processing pipelines and the post-processing utilities. We finish the paper with conclusions and future developments.

## 2. The software ecosystem

The neuroimaging open software ecosystem was born more than 10 years ago, with brain image processing tools such as Statistical Parametric Mapping (SPM12) (Ashburner, 2012), the FMRIB Software Library (FSL) (Jenkinson et al., 2012), AFNI (Analysis of Functional NeuroImages) (Cox, 2012), and FreeSurfer (Fischl, 2012). These libraries were developed by universities and government institutions, and published under open-source or free-software licenses. They have boosted the neuroimaging research, acting as a seed to a now flourishing software ecosystem with new actors appearing lately, e.g., Advanced Normalization Tools (ANTs) (Avants et al., 2014), PET partial volume correction (PETPVC) (Thomas et al., 2016), and MRtrix (Tournier et al., 2012).

As a programming language, Python is becoming very popular. Its supporting community of users and developers is making a great effort to spread good practices of the development process. As a result, there is a booming variety of libraries, guidelines, documentation, development tools, software testing, and continuous integration. A broad collection of tools is available, ranging from general numerical libraries^5^, to specific applications such as machine learning in Scikit-Learn (Pedregosa et al., 2011), or deep neural networks in Tensorflow^6^.

In the neuroscientific field, a group of projects have joined in a community of practice called Nipy^7^. From Nipy, it's possible to find projects to access different neuroimaging file formats^8^, diffusion brain MRI with Dipy (Garyfallidis et al., 2014), statistical learning and fMRI with Nilearn (Abraham et al., 2014) as well as building processing pipelines with Nipype, and others (Gramfort et al., 2013).

Nipype allows to interact and combine tools from different software packages, some already mentioned before, facilitating faster data processing by running the workflows in parallel on many cores/machines. Nipype makes your analyses easily reproducible allowing to share your processing workflows with the community, it also captures the pipelines provenance information in a formal and rigorous way, and allows to restart the pipelines if something has gone wrong. It already presents examples of processing workflows for many imaging modalities. However, it is still a challenge for a non-expert programmer to prepare the data, programme the data selection, configure the workflow, and run it. Built on top of Nipype, Pypes provides workflows for multimodal brain MRI and PET/MRI. We verified the correct working of these pipelines over our clinical dataset of more than 400 subjects and the COBRE database. Pypes uses Nipype as interface to all the command line and Matlab neuroimaging tools. It presents a software structure with a clear way to concatenate and attach new workflows, offering a simple way to express the input data folder structure, and easily configure each node. The output of the workflows are structured in the same way as the input folder structure, and the output file name conventions are homogeneous and clear. The users would have to structure their data following recommendations and use the provided examples of Python functions to build their own pipeline.

## 3. Data preparation and pipeline configuration

Pypes expects neuroimage files to be in the NifTI format. Currently, a good tool to convert from DICOM to NifTI is dcm2niix^9^.

One practical feature that Pypes adds to Nipype is the management of file input and output. The file input selection system is clearer and more flexible than that of NiPype using the parametric file paths provided by the hansel package^10^. The output will have the same folder structure as the input and the naming convention is uniform across the pipelines.

The main nodes in the pipelines are configurable through a configuration file. We recommend using the YAML (https://en.wikipedia.org/wiki/YAML) format for this file. To change the default value of a node parameter one has to add to the configuration file an entry for the value you want. For example, let's say we have a spm.Normalize12 node named anat_warp in one of the workflows. We want to set the value of the parameter bias_regularization to 0.1. We have to add an entry to the configuration file as:

anat_warp . b i a s _ r e g u l a r i z a t i o n : 0 . 1

Pypes includes in its source code an example of configuration file with the main node settings and explanations.

## 4. Pre-processing methods

Each image modality has certain kinds of artifacts and noise, such that specific correction procedures are needed. Multimodal information requires data fusion. Here, we explain the main features of each pre-processing pipeline.

### 4.1. Anatomical MRI

**Figure 1:**
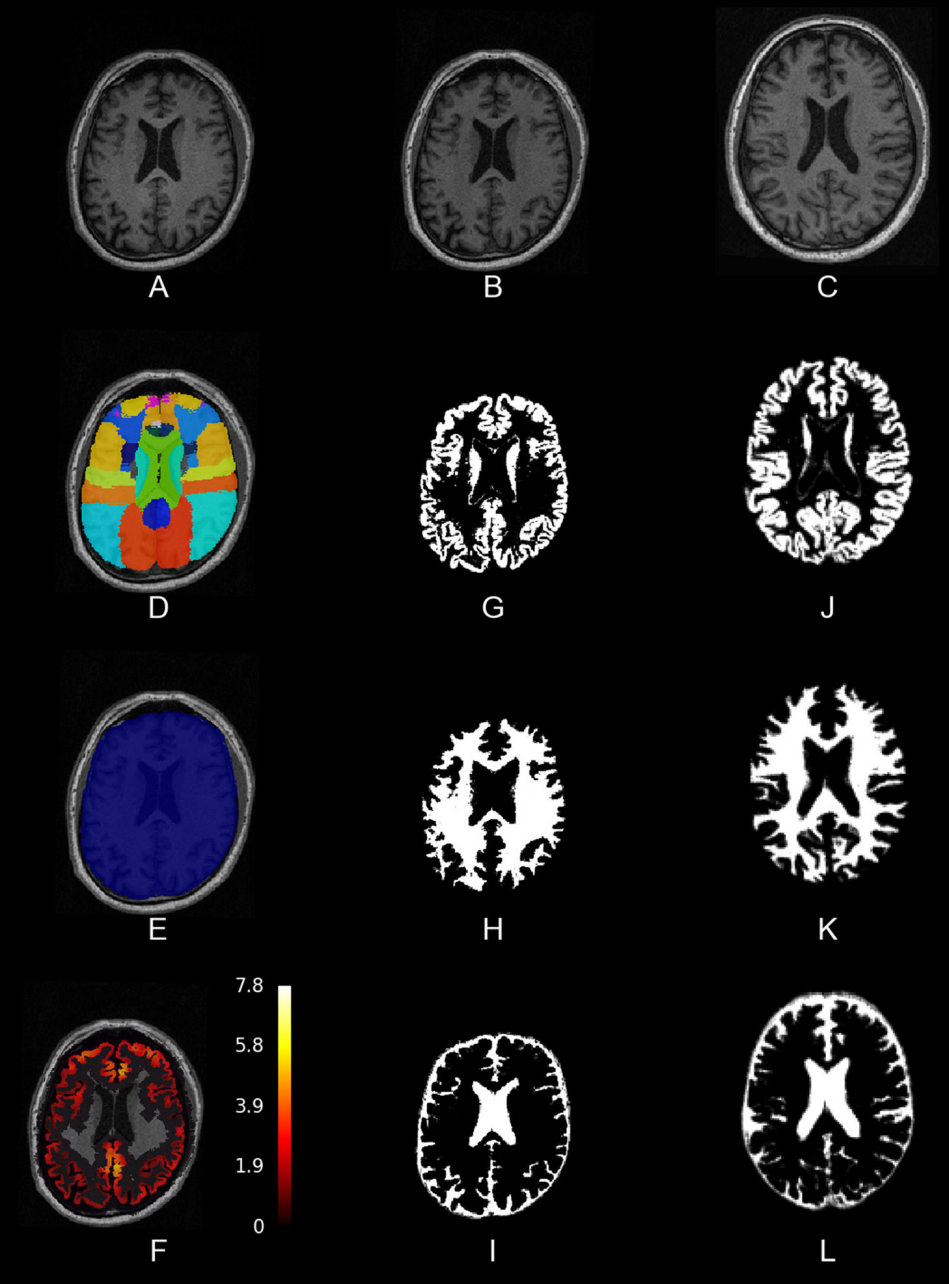
**Slices of one example of the anatomical pipeline on one sample image**. **(A)** The raw MPRAGE image, **(B)** the bias-field corrected MPRAGE, **(C)** the bias-field corrected MPRAGE in MNI space, **(D)** the Hammers atlas in anatomical space, **(E)** a brain mask, and **(F)** the result from the cortical thickness pipeline, **(G–I)** gray matter, white matter and cerebro-spinal fluid (tissue segmentations), and **(J–L)** tissue segmentations in MNI space.

First, we needed to process structural T1-weighted MRI images. We prepared a pipeline that first performs a bias-field correction using ANTs' N4BiasFieldCorrection (Tustison et al., 2010). Then it segments the brain tissues and registers the image to a standard template space (MNI) with the SPM12 New Segment tool. Finally, it creates a brain mask based on the tissue segmentations. In addition, we needed to warp an atlas, or any other image in the SPM12 standard space, to the subject's native space for further analyses. In Figure 1, we present an image output of this pipeline. It is implemented in pypes.anat.preproc.spm_anat_preprocessing.

### 4.2. Positron emission tomography

The computational analysis in the reference clinical study requires pre-processing of FDG-PET images that had been acquired simultaneously with MRI data (Savio et al., 2017). Pypes offers two main pipelines for PET data, one with and the other without involving T1-weighted MRI. Both pipelines use SPM12 Normalize to warp PET images to the MNI space. The latter option requires a group-template pipeline, where a group template is created from all subjects, and then all PET images are normalized to this group template.

**Figure 2:**
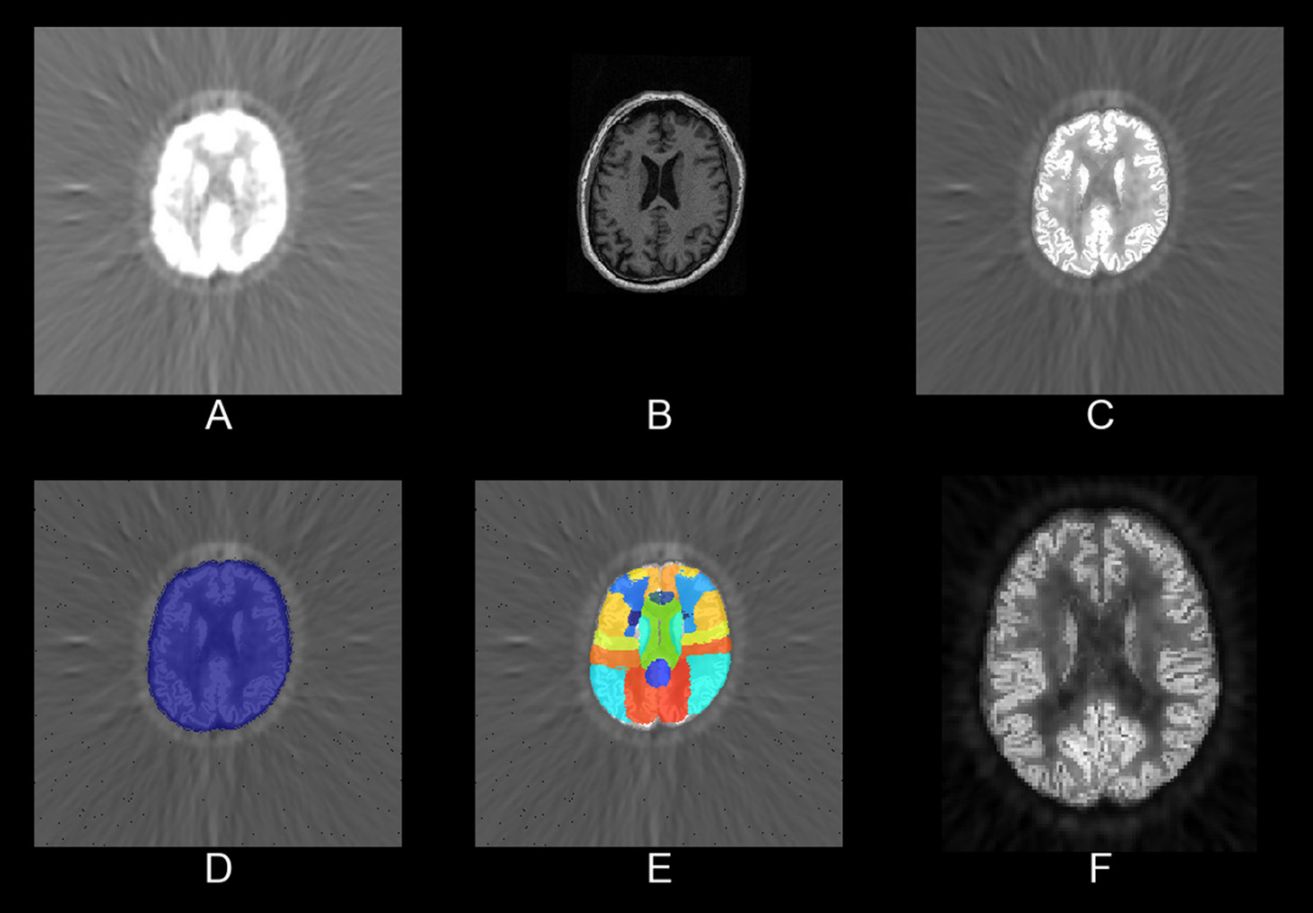
**Slices of partial results from one sample processed by the PET/MR pipeline**. **(A)** The raw FDG-PET, **(B)** the MPRAGE, **(C)** the partial volume corrected PET, **(D)** a brain mask, **(E)** the Hammers' atlas, and **(F)** the PVC PET in MNI space.

The PET/MR pipeline also applies PVC and normalizes structural MRI data to the PET space. It is implemented in pypes.pet.mrpet.spm_mrpet_preprocessing. In Figure 2, we present an image output of this pipeline.

#### 4.2.1. Partial volume correction (PVC)

One of the main issues with PET is signal degradation caused by partial volume effects (PVE). PVC methods improve the quantitative accuracy of PET data by compensating for the noise owing to a limited scanner resolution (Erlandsson et al., 2012), specially in the atrophied brains. Generally, brain anatomical information from MRI is used here. The recently published PETPVC (Thomas et al., 2016) library^11^ provides eight core methods of PVC in an open source tool.

### 4.3. Functional MRI

The referred clinical study investigates functional connectivity, so we implemented a configurable rsfMRI data processing pipeline. For this, we need anatomical localization for nuisance corrections based on tissue signal, so this pipeline is connected to the MPRAGE processing pipeline (Section 4.1). The first part of this pipeline, implemented in pypes.fmri.clean.fmri_cleanup_wf, trims the first 6 s from the data, carries out slice-time correction based on SPM12 SliceTiming, correction for motion with Nipy's SpaceTimeRealigner (Roche, 2011), co-registration of the tissues from anatomical space to fMRI space, correction for nuisances extracted from time-course SNR (TSNR) estimation, artifact detection from Nipype's rapidART, motion correction, signal component regression from different tissues (CSF, WM, and/or GM), and optionally, global signal regression. The trends detected from these filters are regressed out from the fMRI data. Each of these corrections are optional and configurable. After the nuisance correction step, a bandpass time filter is applied to extract resting-state frequencies and the data is spatially smoothed. In the second step of this pipeline, implemented in pypes.fmri.warp.spm_warp_fmri_wf, the main outputs of the first are warped to MNI using SPM12 Normalize.

### 4.4. Diffusion-tensor MRI (DTI)

DTI may be useful to support or extend findings of metabolic and functional connectivity. We provide a pipeline that performs DTI correction and pre-processing, tensor-fitting, and tractography. This pipeline is implemented in pypes.dmri.dti.attach_spm_fsl_dti_preprocessing. First is uses FSL Eddy (Andersson and Sotiropoulos, 2016) for Eddy currents and motion correction. Then Non-local Means (Coupe et al., 2008) is used from Dipy for image de-noising with a Rician filter. This pipeline also estimates motion statistics with Nipype's RapidArt for *post-hoc* quality check, co-registers the anatomical image to diffusion space, and rotates the b-vectors based on motion estimation from Eddy. Optionally, it will warp an atlas to diffusion space (for further tractography).

**Figure 3:**
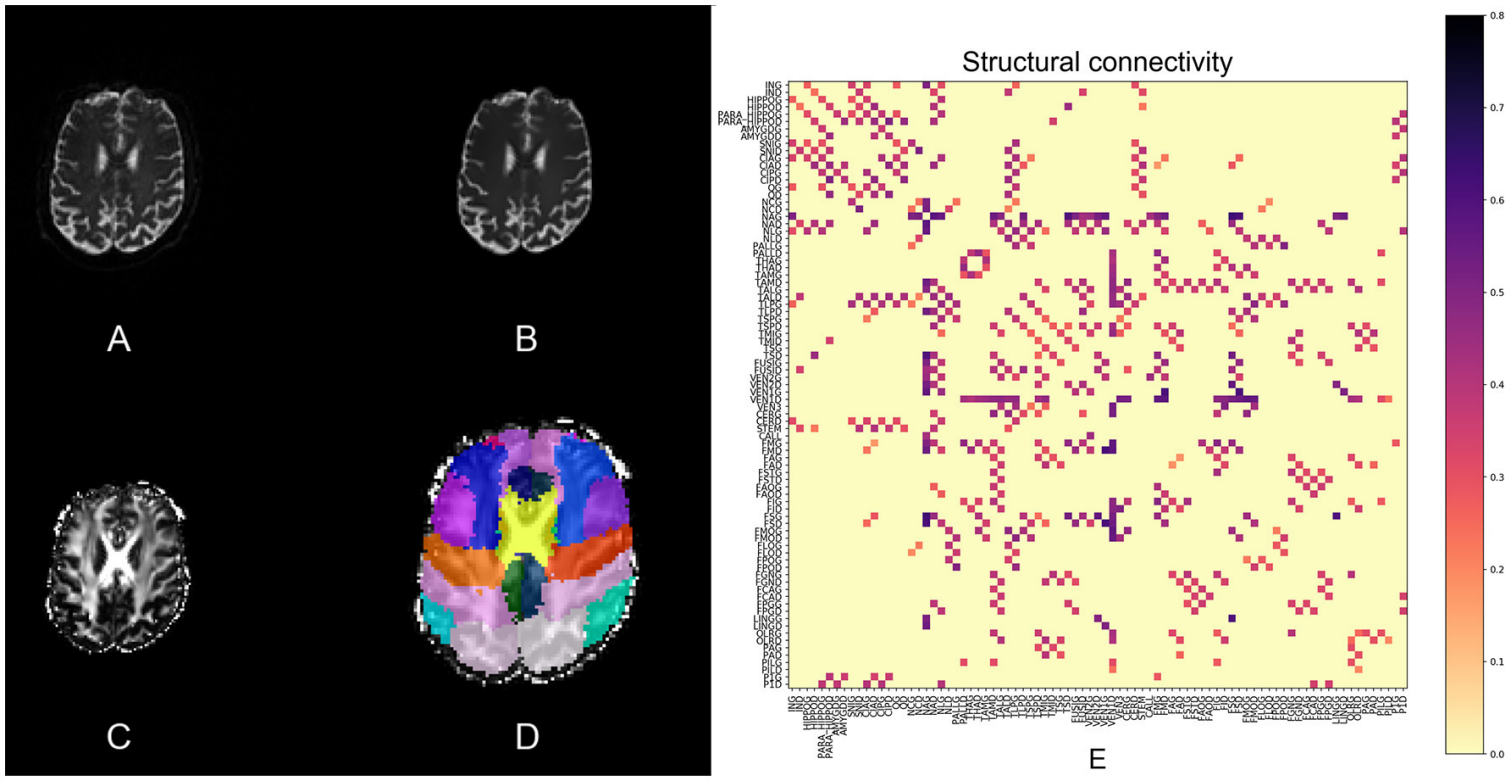
**Slices of one example from the DTI processing pipeline: (A)** the raw DTI (*b*-value = 0) image, **(B)** the Eddy-currents corrected and nl-means denoized image, **(C)** the FA image, and **(D)** the atlas in DTI space. **(E)** Shows the structural connectivity matrix calculated with Camino.

An extra pipeline implemented in pypes.dmri.camino.camino_tractography uses Camino (Friman et al., 2006) to calculate Fractional Anisotropy (FA) and perform ROI-to-ROI deterministic tractography using Track (Basser et al., 1994). The tractography pipeline exports two connectivity matrices: one with the number of tracts and the other with average tract FA values, for each pair of ROIs. Figure 3 illustrates the DTI pipeline, including the connectivity matrix.

## 5. Post-processing methods

Pypes offers extra utilities and shorter *post-hoc* pipelines. It provides an Independent Component Analysis (ICA) interface to use Nilearn's CanICA and DictLearning against individual or group of fMRI data. These pipelines are implemented in pypes.postproc.decompose. For the analysis of ICA results and resting state networks, we implemented different similarity measures in pypes.ica.spatial_maps, including Goodness of fit (Zhou et al., 2010), Pearson's correlation, and many others. A cortical thickness pipeline based on ANTs (Tustison et al., 2014) is implemented in pypes.anat.cortex.

We created plotting utilities to visualize and publish the pre- and post-processing results. In pypes.ica.plotting, we implemented a series of methods to plot ICA spatial maps and loading coefficients from Nilearn and GIFT^12^.

## 6. Conclusion and future work

We have published Pypes, a tool that allows an easy configuration of workflows for neuroimage analysis using resources from state of the art open source libraries, while including new handy facilities for input/output data configuration and plotting. Pypes gives support to the growing community of researchers having access to hybrid PET/MRI scanners which require complex proprocessing and analysis of multimodal imaging data. For more details on how to use Pypes, check the documentation on http://neuro-pypes.readthedocs.io/.

Some aspects of PET/MRI processing are still to be considered in Pypes. A synchronized acquisition allows the use of MRI information for attenuation correction (AC) of PET images, though there is no consensus on the best algorithm (Cabello et al., 2016; Mehranian et al., 2016; Ladefoged et al., 2017). Future works will include the proposed AC algorithms in the PET/MRI pipelines of Pypes. Also other non-linear registration tools and DTI tractography methods should be added. Easy connection to machine learning libraries such as Nilearn and scikit-learn would allow further automatization of analyses and creation of predictive models for e.g., disease detection.

## Author contributions

AS, MS, and IY did the software requirements, design; MS and AS did the programming; MG contributed in the writing process. All authors contributed in drafting the work and revising it critically for important intellectual content; all authors give final approval of the version to be published; all authors agree to be accountable for all aspects of the work in ensuring that questions related to the accuracy or integrity of any part of the work are appropriately investigated and resolved.

### Conflict of interest statement

The authors declare that the research was conducted in the absence of any commercial or financial relationships that could be construed as a potential conflict of interest.

## Footnotes

^1^http://nipype.readthedocs.io/

^2^http://fcp-indi.github.io/

^3^http://www.rfmri.org/DPARSF

^4^http://fcon_1000.projects.nitrc.org/indi/retro/cobre.html.

^5^http://www.scipy.org/

^6^https://www.tensorflow.org

^7^http://nipy.org/

^8^http://nipy.org/nibabel.

^9^https://github.com/rordenlab/dcm2niix

^10^https://github.com/alexsavio/hansel.

^11^https://github.com/UCL/PETPVC.

^12^http://mialab.mrn.org/software/gift/.
